# Abnormal folate metabolism causes age‐, sex‐ and parent‐of‐origin‐specific haematological defects in mice

**DOI:** 10.1113/JP276419

**Published:** 2018-08-15

**Authors:** Nisha Padmanabhan, Katerina Menelaou, Jiali Gao, Alexander Anderson, Georgina E. T. Blake, Tanya Li, B. Nuala Daw, Erica D. Watson

**Affiliations:** ^1^ Department of Physiology, Development and Neuroscience University of Cambridge Cambridge UK; ^2^ Centre for Trophoblast Research University of Cambridge Cambridge UK

**Keywords:** extramedullary haematopoiesis, macrocytic anemia, MTRR, parental effect, sexual dimorphism, late‐onset, mice

## Abstract

**Key points:**

- Folate (folic acid) deficiency and mutations in folate‐related genes in humans result in megaloblastic anaemia.
- Folate metabolism, which requires the enzyme methionine synthase reductase (MTRR), is necessary for DNA synthesis and the transmission of one‐carbon methyl groups for cellular methylation.
- In this study, we show that the hypomorphic *Mtrr^gt/gt^* mutation in mice results in late‐onset and sex‐specific blood defects, including macrocytic anaemia, extramedullary haematopoiesis and lymphopenia.
- Notably, when either parent carries an *Mtrr^gt^* allele, blood phenotypes result in their genetically wildtype adult daughters, the effects of which are parent specific.
- Our data establish a new model for studying the mechanism of folate metabolism in macrocytic anaemia aetiology and suggest that assessing parental folate status might be important when diagnosing adult patients with unexplained anaemia.

**Abstract:**

The importance of the vitamin folate (also known as folic acid) in erythrocyte formation, maturation and/or longevity is apparent since folate deficiency in humans causes megaloblastic anaemia. Megaloblastic anaemia is a type of macrocytic anaemia whereby erythrocytes are enlarged and fewer in number. Folate metabolism is required for thymidine synthesis and one‐carbon metabolism, though its specific role in erythropoiesis is not well understood. Methionine synthase reductase (MTRR) is a key enzyme necessary for the progression of folate metabolism since knocking down the *Mtrr* gene in mice results in hyperhomocysteinaemia and global DNA hypomethylation. We demonstrate here that abnormal folate metabolism in mice caused by *Mtrr^gt/gt^* homozygosity leads to haematopoietic phenotypes that are sex and age dependent. Specifically, *Mtrr^gt/gt^* female mice displayed macrocytic anaemia, which might be due to defective erythroid differentiation at the exclusion of haemolysis. This was associated with increased renal *Epo* mRNA expression, hypercellular bone marrow, and splenic extramedullary haematopoiesis. In contrast, the male response differed since *Mtrr^gt/gt^* male mice were not anaemic but did display erythrocytic macrocytosis and lymphopenia. Regardless of sex, these phenotypes were late onset. Remarkably, we also show that when either parent carries an *Mtrr^gt^* allele, a haematological defect results in their adult wildtype daughters. However, the specific phenotype was dependent upon the sex of the parent. For instance, wildtype daughters of *Mtrr^+/gt^* females displayed normocytic anaemia. In contrast, wildtype daughters of *Mtrr^+/gt^* males exhibited erythrocytic microcytosis not associated with anaemia. Therefore, abnormal folate metabolism affects adult haematopoiesis in an age‐, sex‐ and parent‐specific manner.

## Key points

- Folate (folic acid) deficiency and mutations in folate‐related genes in humans result in megaloblastic anaemia.
- Folate metabolism, which requires the enzyme methionine synthase reductase (MTRR), is necessary for DNA synthesis and the transmission of one‐carbon methyl groups for cellular methylation.
- In this study, we show that the hypomorphic *Mtrr^gt/gt^* mutation in mice results in late‐onset and sex‐specific blood defects, including macrocytic anaemia, extramedullary haematopoiesis and lymphopenia.
- Notably, when either parent carries an *Mtrr^gt^* allele, blood phenotypes result in their genetically wildtype adult daughters, the effects of which are parent specific.
- Our data establish a new model for studying the mechanism of folate metabolism in macrocytic anaemia aetiology and suggest that assessing parental folate status might be important when diagnosing adult patients with unexplained anaemia.

## Introduction

The importance of folate metabolism during erythropoiesis is demonstrated by the appearance of megaloblastic anaemia in folate‐deficient humans. Megaloblastic anaemia is a type of macrocytic anaemia characterized by a low red blood cell (RBC) count, erythrocytes that are macrocytic, fewer immature RBCs (e.g. reticulocytes) and fewer platelets (Koury & Ponka, 2004). A critical characteristic of megaloblastosis is the asynchronous maturation of the cytoplasmic and nuclear compartments of erythroid progenitors leading to abnormal morphology. Folate is essential for many biochemical reactions including *de novo* synthesis of purines and pyrimidines (Koury & Ponka, 2004). It also acts as a carrier of one‐carbon methyl groups destined to methylate specific cellular targets. These include DNA (Jacob *et al*. 1998) and proteins, such as histones (Ghandour *et al*. 2002), the methylation of which is required to regulate chromatin structure and thus gene expression. Although the mechanism is unclear, the high rate of proliferation during erythropoiesis might make erythroid progenitors particularly susceptible to genomic instability (Menzies *et al*. 1966; Wickramasinghe *et al*. 1968; Yoshida *et al*. 1968; Koury *et al*. 1997). Additionally, epigenomic instability caused by global hypomethylation in the case of abnormal folate metabolism (Padmanabhan *et al*. 2013) might cause gene misexpression leading to abnormal differentiation or increased cell death of erythrocyte precursors.

Few animal models exist for the pathophysiological study of macrocytic anaemia related to folate deficiency. Mice fed a folate‐free amino acid‐based diet exhibit pancytopenia but lack macrocytic cells (Bills *et al*. 1992). However, when folate‐deficient mice are infected with the Friend leukaemia virus, which increases the proliferation rate of erythrocyte precursors, megaloblastic erythrocytosis is accentuated (Koury & Horne, 1994). Others have shown that when intestinal cells are unable to absorb folate, such as in *Slc46a1^−/−^* mice that lack the folate carrier PCFT, a severe case of macrocytic normochromic anaemia results (Salojin *et al*. 2011). Supplementation of folinic acid, 5‐methyltetrahydrofolate (5‐methyl‐THF) or folic acid promotes the survival of *Slc46a1^−/−^* mice by improving RBC parameters (Salojin *et al*. 2011). Similarly, mutations in the human *SLC46A1* gene lead to folate malabsorption syndrome and megaloblastic anaemia (Erlacher *et al*. 2015). SLC19A1 (also known as reduced folate carrier 1, RFC1) is a protein essential for folate transport in mammalian cells. A knockout of the mouse *Slc19a1* gene causes embryonic lethality (Gelineau‐van Waes *et al*. 2008). However, when maternal folate supplementation was provided, *Slc19a1^−/−^* pups survived until birth, but died within 12 days postpartum due to a failure of haematopoietic organs (Zhao *et al*. 2001). Mutations in human genes associated with the metabolism of folate including methionine synthase (*MTR*) (Gulati *et al*. 1996; Leclerc *et al*. 1996) and methionine synthase reductase (*MTRR*) (Zavadakova *et al*. 2002; Vilaseca *et al*. 2003) also lead to megaloblastic anaemia. However, mice with mutations in the *Mtr*, *Mtrr* or *Mthfr* genes are not well characterized for haematological defects.

During the metabolism of folate, MTR is an enzyme responsible for exclusively transferring the methyl‐group from 5‐methyl‐THF to homocysteine to form methionine and tetrahydrofolate (Shane & Stokstad, 1985). Methionine is a precursor to *S*‐adenosyl methionine (*S*‐AdoMet), which serves as a methyl‐donor to cellular substrates including proteins, RNA and DNA (Wainfan & Maschio, 1975; Jacob *et al*. 1998; Ghandour *et al*. 2002), a process that requires methyltransferases. MTRR is a key enzyme necessary for the activation of MTR through the reductive methylation of its vitamin B_12_ cofactor (Yamada *et al*. 2006). Consequently, progression of the folate and methionine cycles requires MTRR enzymatic activity. A hypomorphic mutation of the mouse *Mtrr* gene (denoted as *Mtrr^gt^*) disrupts folate metabolism by creating a ‘methyl trap’ as determined by an increase in plasma homocysteine and liver 5‐methyl‐THF concentrations, a reduction in plasma methionine and heart *S*‐adenosyl methionine/*S*‐adenosyl homocysteine ratio, and global DNA hypomethylation (Elmore *et al*. 2007; Padmanabhan *et al*. 2013). Accordingly, the *Mtrr^gt^* mouse line is ideal for exploring the effects of abnormal folate metabolism on haematopoietic lineage. Here, we show that *Mtrr^gt/gt^* mice display haematological phenotypes, such as macrocytic anaemia, that are dependent upon sex and age.

It is well established that maternal or paternal folate deficiency in humans and mice increases the risk of developmental phenotypes in their offspring (MRC Vitamin Study Research Group, 1991; Lambrot *et al*. 2013), the most famous example of which is neural tube defects. However, little attention has been given to the parental effects of abnormal folate uptake or metabolism on adult‐onset disease in their offspring, including megaloblastic anaemia. Therefore, we sought to explore whether an *Mtrr^gt^* allele in female or male mice was sufficient to cause haematological abnormalities in their wildtype daughters (i.e. the F1 generation). Our study shows that wildtype F1 female mice display differential haematological defects, such as anaemia, depending on the *Mtrr* genotype of their mother or father. Altogether, we demonstrate that abnormal folate metabolism as caused by the *Mtrr^gt^* allele affects haematopoiesis in an age‐, sex‐ and parent‐specific manner.

## Methods

### Ethical approval

This research was regulated under the Animals (Scientific Procedures) Act 1986 Amendment Regulations 2012 following ethical review by the University of Cambridge Animal Welfare and Ethical Review Body. The experiments were carried out according to the principles and regulations described by Grundy (2015).

### Mice and diet

The *Mtrr^gt^* mouse line was originally generated by a gene‐trap (gt) insertion as previously described (Padmanabhan *et al*. 2013). Since the *Mtrr^gt^* mutation was originally backcrossed into a C57Bl/6 mouse genetic background (Padmanabhan *et al*. 2013), C57Bl/6 mice (The Jackson Laboratory, Bar Harbor, ME, USA) were used as controls in all experiments and were maintained in‐house but separately from the *Mtrr^gt^* mouse line. *Mtrr^gt/gt^* mice were generated by *Mtrr^gt/gt^* intercrosses. The maternal or paternal effect of the *Mtrr^gt^* allele was determined by analysis of *Mtrr^+/+^* females derived from either *Mtrr^+/gt^* females crossed to C57Bl/6 males or *Mtrr^+/gt^* males crossed to C57Bl/6 females, respectively. All mice assessed were virgins. PCR genotyping was performed as previously described (Padmanabhan *et al*. 2013). Mice were fed a normal breeding diet (Rodent No. 3 breeding chow, Special Diet Services, Essex, UK) *ad libitum* from weaning onward. A full dietary breakdown of the chow was previously described (Padmanabhan *et al*. 2013). Mice were killed for tissue collection by cervical dislocation.

### Haematological profiling

Two age groups of virgin mice were analysed: female and male mice at 7 weeks of age, and female and male mice averaging 5 months of age (*n* = 3–5 mice per sex per age group). The parental effect of the *Mtrr^gt^* mutation was determined in 5‐month‐old wildtype F1 female mice (*n* = 6–7 mice per pedigree). Age‐ and sex‐matched C57Bl/6 mice were used as controls (*n* = 5–6 mice per experiment). Peripheral blood was collected with a 25‐gauge needle through direct cardiac puncture after cervical dislocation. Fresh blood samples were placed into ethylene diamine tetraacetic acid (EDTA)‐coated tubes and sent for machine blood count analysis at Central Diagnostic Services, Clinical Pathology Laboratory, Cambridge Veterinary School, University of Cambridge. The profiles for C57Bl/6 mice were comparable to standard values (Russell & Meier, 1968). Blood smears were prepared and analysed to confirm the findings. The reticulocyte production index (RPI) was calculated as previously described (Salojin *et al*. 2011). Briefly, the percentage of reticulocytes in peripheral blood was multiplied by the ratio of peripheral blood haematocrit (HCT) to the mean HCT in the C57Bl/6 group (%Reticulocytes × [HCT/(mean C57Bl/6 HCT)]). The RPI was further adjusted for longer lifespan of reticulocytes in mice with low HCT by dividing RPI values by the reticulocyte maturation index (Prouty, 1979), which was arbitrarily set to 1 for blood samples with HCT greater than 41.0%, and to 1.5 for samples with HCT values within the 31.0–40.9% range (Salojin *et al*. 2011).

### Bone marrow isolation

Bone marrow cells were isolated from female femurs (*n* = 3–4 mice per pedigree) based on a published protocol (Swamydas & Lionakis, 2013). Briefly, muscles were removed from femur and humerus bones, which were then washed in 1x phosphate buffered saline (PBS). The epiphyses of the bones were removed with a scalpel and, using a 25‐gauge needle, the bone marrow cells were flushed out with 1x PBS. The inner surface of the bones was scraped with the needle to ensure efficient removal of cells. The cell suspension was centrifuged at 300 ×*g* for 7 min at 4°C and the resulting pellet was frozen in liquid nitrogen and stored at −80°C.

### Histology

Tissue was dissected from 3–6 mice per sex and per genotype/pedigree. Femurs with muscle removed were fixed in 10% neutral buffered formalin for 48 h at 4°C and washed in 1x PBS. The bones were treated with Decalcifying Solution‐Lite (Sigma‐Aldrich, Gillingham, UK) before preparing for paraffin embedding. Spleens were fixed in 4% paraformaldehyde in 1x PBS and prepared for paraffin embedding using standard techniques. Paraffin blocks were sectioned to 7 μm. Tissues were stained with haematoxylin and eosin (H & E) using standard procedures.

### Immunohistochemistry and Prussian blue stain

Paraffin‐embedded tissue sections were de‐waxed, rehydrated and washed in 1x PBS. For immunohistochemistry staining, tissue sections were incubated in 3% H_2_O_2_ in 1x PBS for 30 min, treated with trypsin tablets (Sigma‐Aldrich) for 10 min, and then incubated with blocking serum (5% donkey serum, 1% bovine serum albumin in 1x PBS) for 1 h. Tissue was incubated in rabbit anti‐mouse Ki67 (Abcam, Cambridge, UK, cat. no. ab15580, RRID:AB_443209) diluted to 1:100 in blocking serum overnight at 4°C, and then in donkey anti‐rabbit IgG conjugated to horseradish peroxidase (Abcam cat. no. ab6802, RRID:AB_955445) diluted to 1:300 in blocking serum for 1 h at room temperature. Peroxidase substrate reactions were conducted with DAB (3,3′‐diaminobenzidine) chromagen substrate kit (Abcam cat. no. ab64238) according to the manufacturer's instructions. For Prussian blue stain, tissue was placed in a 50:50 working solution of 0.01 M potassium ferrocyanide and 0.6% hydrochloric acid for 15 min. Sections were counterstained with Nuclear Fast Red (Sigma‐Aldrich) before dehydration, clearing and mounting in DPX medium (Sigma‐Aldrich).

### RNA extraction and quantitative reverse transcription PCR (RT‐qPCR)

Tissue was homogenized using Lysing Matrix D beads (MP Biomedicals, Carlsbad, CA, USA). Total RNA extraction was completed using Trizol (Sigma‐Aldrich), the GenElute Mammalian Total RNA Miniprep Kit (Sigma‐Aldrich), or AllPrep DNA/RNA Mini Kit (QIAGEN, Manchester, UK) according to the manufacturer's instructions. All extracts were treated with DNase I (Thermo Scientific, Waltham, MA, USA). Reverse‐transcription reactions were performed with the RevertAid H Minus First Strand complementary DNA (cDNA) Synthesis Kit (Thermo Scientific) by using 2–5 mg of RNA in a 20 mL reaction according to the manufacturer's instructions. PCR amplification of *Runx1b*, *Hbb*, *Ftl1* and *Hamp1* transcripts was conducted using MESA SYBR Green qPCR MasterMix Plus (Eurogentec, Liege, Belgium). PCR amplification of *Epo* transcripts was conducted using FastStart TaqMan Probe master mix (Roche, Basel, Switzerland). A DNA Engine Opticon 2 thermocycler (BioRad, Hercules, CA, USA) was used in both cases. Transcript levels were normalized to *Gapdh*, *Actb* and/or *Hprt* RNA levels, and the fold change was quantified using the standard curve method (*Runx1b*, *Hbb* and *Hamp1*) or the comparative Ct method (*Epo*). cDNA levels in C57Bl/6 tissue were normalized to 1. Experiments were conducted in duplicate or triplicate with at least three biological replicates. Primer sequences were as follows: *Actb* (forward [F]) 5′‐CCC TAA GGC CAA CCG TGA A, (reverse [R]) 5′‐CAG CCT GGA TGG CTA CGT ACA; *Epo* (F) 5′‐TCT GCG ACA GTC GAG TTC TG, (R) 5′‐CTT CTG CAC AAC CCA TCG T; *Ftl1* (F) 5′‐CCT CGC TGC CTT CAG CTC, (R) 5′‐ AAA GAA GCC CAG AGA GAG GT; *Gapdh* (F) 5′‐CAT GGC CTT CCG TGT TCC T, (R) 5′‐GCG GCA CGT CAG ATC CA (Gillich *et al*. 2012); *Hamp1* (F) 5′‐GAT GGC ACT CAG CAC TCG, (R) 5′‐GCT GCA GCT CTG TAG TCT GTC T (Patel *et al*. 2012); *Hbb* (F) 5′‐ GTC TCT GGC CTG TGG GGA AA, (R) 5′‐CAA CCA GCA GCC TGC CC; *Hprt* (F) 5′‐TCC TCC TCA GAC CGC TTT T, (R) 5′‐ CCT GGT TCA TCA TGC CTA ATC; *Runx1b* (F) 5′‐ CCT CCG GTA GTA ATA AAG GCT TCT G, (R) 5′‐CCG ATT GAG TAA GGA CCC TGA A (Challen & Goodell, 2010).

### Total homocysteine concentrations

Total homocysteine concentrations in plasma were simultaneously measured by the Biochemical Genetics Unit, Department of Clinical Biochemistry, Cambridge University Hospitals NHS Foundation Trust using underivatized liquid chromatography tandem‐mass spectrometry (LC‐MS/MS) (Waters ACQUITY liquid chromatography system and Quattro Premier mass spectrometer) operated in electrospray ionization positive mode as was previously described in detail (Padmanabhan *et al*. 2013).

### Equipment and software

A Zeiss AXIO Imager A.1 light microscope with a Zeiss AxioCam MRc5 camera and AxioVision 4.7.2 imaging software program (Zeiss, Oberkochen, Germany) were used to obtain cell and tissue images. Cell counts, Prussian blue staining intensity and other histological measurements were performed using ImageJ (64‐bit) software (NIH, Bethesda, MD, USA). Graphs were generated using GraphPad Prism 6 software (Graphpad Software Inc., La Jolla, CA, USA).

### Statistical analysis

Statistical analyses were performed using GraphPad Prism 6 software. Data were analysed using independent Student's *t* tests or ordinary one‐way ANOVA with Tukey multiple comparison tests. *P* values less than 0.05 were considered significant.

## Results

### *Mtrr^gt/gt^* female mice display macrocytic anaemia

**Figure 1:**
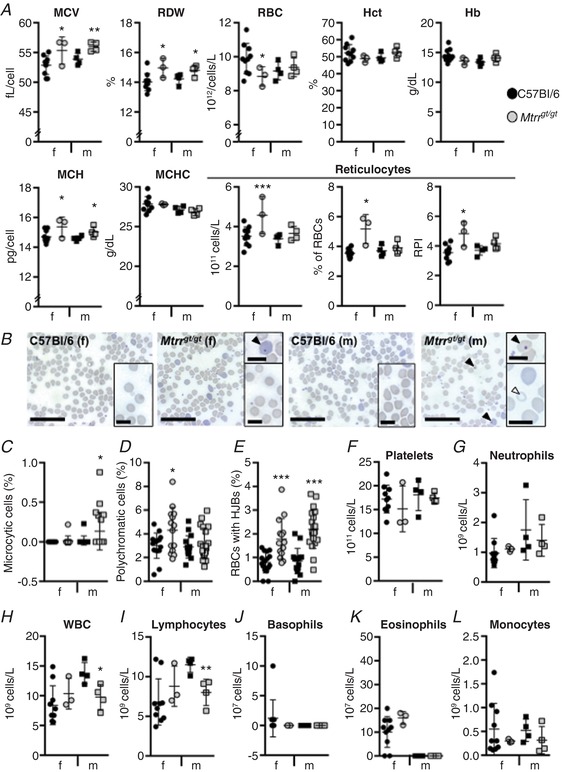
Sexually dimorphic haematological defects in peripheral blood from *Mtrr^gt/gt^* mice compared to C57Bl/6 controls at 5 months of age Haematological profiles of peripheral blood from C57Bl/6 control mice (black symbols) *versus Mtrr^gt/gt^* mice (grey symbols) at 5 months of age. Graphical data are presented as means ± SD. Blood from female (f, circles) and male (m, squares) mice was assessed. *n* = 3–10 mice per sex and genotype. *A*, graphs indicating erythroid cell characteristics of C57Bl/6 and *Mtrr^gt/gt^* mice. MCV, mean corpuscular volume; RDW, red blood cell distribution width; RBC, red blood cells; Hct, haematocrit; Hb, haemoglobin; MCH, mean corpuscular haemoglobin; MCHC, mean corpuscular haemoglobin concentration; RPI, reticulocyte production index. *B*, representative images of peripheral blood smears from C57Bl/6 and *Mtrr^gt/gt^* mice. Blood from female (f) and male (m) mice was assessed. Black arrowheads indicate RBCs with Howell–Jolly bodies. White arrowhead indicates a microcytic cell. Scale bars: 10 μm; insets, 3 μm. *C* **–** *E*, graphs indicating the percentage of RBCs that were microcytic (*C*), polychromatic (*D*) or contained Howell–Jolly bodies (HJBs, *E*) in peripheral blood smears of C57Bl/6 and *Mtrr^gt/gt^* mice. *F* **–** *L*, graphs indicating the number of platelets (*F*), neutrophils (*G*), white blood cells (WBC, *H*), lymphocytes (*I*), basophils (*J*), eosinophils (*K*) and monocytes (*L*) in the peripheral blood of C57Bl/6 and *Mtrr^gt/gt^* mice. Statistical analysis: unpaired *t* tests were performed to independently compare C57Bl/6 females to *Mtrr^gt/gt^* females or C57Bl/6 males to *Mtrr^gt/gt^* males. ^*^ *P* < 0.05, ^**^ *P* < 0.01, ^***^ *P* < 0.001. [Color figure can be viewed at http://wileyonlinelibrary.com]

To determine whether abnormal folate metabolism leads to haematological anomalies in mice, peripheral blood was collected from 5‐month‐old *Mtrr^gt/gt^* mice for profiling and compared to similar age C57Bl/6 controls. Both female and male *Mtrr^gt/gt^* mice displayed red blood cell (RBC) distribution widths that were significantly greater than controls (*P* < 0.03) indicating more variable RBC size in the *Mtrr^gt/gt^* mice (Fig. 1 *A* and *B*). The mean corpuscular volumes were also significantly higher than controls (*P* < 0.026; Fig. 1 *A*) indicating macrocytosis. Despite this, a higher frequency of microcytic cells was also detected in peripheral blood smears but only in *Mtrr^gt/gt^* males compared to C57Bl/6 males (Fig. 1 *B* and *C*). Consistent with the broader appearance of macrocytic cells, the mean corpuscular haemoglobin (MCH) level was also significantly higher in female and male *Mtrr^gt/gt^* mice (*P* < 0.03) and the mean corpuscular haemoglobin concentration (MCHC) was unchanged (*P* > 0.19; Fig. 1 *A*). Importantly, *Mtrr^gt/gt^* females (*P* = 0.043) but not *Mtrr^gt/gt^* males (*P* = 0.302) exhibited significantly lower RBC counts compared to C57Bl/6 suggesting sex‐specific anaemia (Fig. 1 *A*). Although haemoglobin levels showed a downward trend in female *Mtrr^gt/gt^* mice compared to C57Bl/6 females (*P* = 0.085), haemoglobin and haematocrit levels were not significantly different in all *Mtrr^gt/gt^* mice assessed (Fig. 1 *A*). Together, these data indicate that *Mtrr^gt/gt^* mice display RBC macrocytosis and, notably, this finding was associated with anaemia only in *Mtrr^gt/gt^* females.

Remarkably, significantly more reticulocytes (RBC precursor cells) were observed in the peripheral blood of *Mtrr^gt/gt^* female mice (*P* = 0.009) and not *Mtrr^gt/gt^* males (*P* = 0.131) compared to C57Bl/6 mice (Fig. 1 *A*). This observation was supported by a higher reticulocyte production index (*P* = 0.003; Fig. 1 *A*) and an increased frequency of polychromatic cells in the peripheral blood smears only in *Mtrr^gt/gt^* females (*P* < 0.05; Fig. 1 *B* and *D*). These data suggest that abnormal folate metabolism in *Mtrr^gt/gt^* females might promote increased formation of erythroid progenitor cells to compensate for the anaemic state or, alternatively, abnormal differentiation of reticulocytes may occur.

### Increased frequency of Howell–Jolly bodies in *Mtrr^gt/gt^* RBCs

Howell–Jolly bodies are erythrocyte micronuclei that fail to be expelled during maturation. Even though Howell–Jolly bodies are present in normal circulating mouse RBCs (Bannerman, 1983), a significantly higher percentage of RBCs containing Howell–Jolly bodies (nuclear fragments) were observed in both *Mtrr^gt/gt^* females and males compared to control C57Bl/6 mice (Fig. 1 *B* and *E*). These bodies are frequently associated with megaloblastic anaemia and folate deficiency in humans (Koury & Ponka, 2004), and may be indicative of altered splenic function (Corazza *et al*. 1990) or impaired erythrocytic maturation.

### *Mtrr^gt/gt^* mice show sex‐specific abnormalities in lymphocyte counts

To explore whether the effects of abnormal folate metabolism were specific to the erythroid lineage, other blood cell populations in peripheral blood were assessed (Fig. 1 *F*–*L*). Myeloid lineages, such as platelets, neutrophils, monocytes, eosinophils and basophils were present in normal numbers in *Mtrr^gt/gt^* male and female mice (Fig. 1 *F*–*L*). Despite this, *Mtrr^gt/gt^* males displayed a significant decrease in lymphocytes compared to control C57Bl/6 males (*P* = 0.005) resulting in an overall decrease in white blood cell (WBC) counts (*P* = 0.013; Fig. 1 *H* and *I*). In contrast, *Mtrr^gt/gt^* females displayed lymphocytes and WBC numbers that were within the normal range (*P* > 0.16; Fig. 1 *H* and *I*). Altogether, these data indicate that the differentiation potential of multiple haematopoietic lineages is influenced by *Mtrr* deficiency in a sex‐specific manner.

### The haematological effects of *Mtrr* deficiency emerge with age

**Figure 2:**
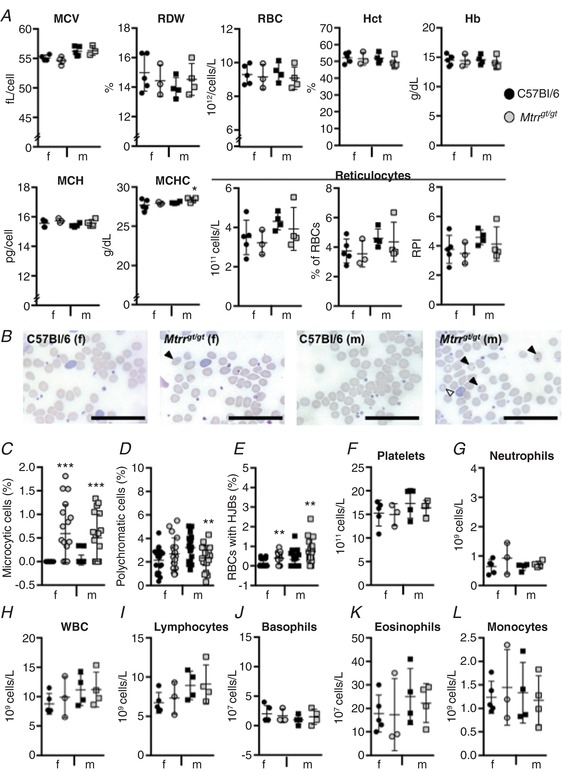
*Mtrr^gt/gt^* mice at 7 weeks have normal peripheral blood haematological profiles Haematological profiles of peripheral blood from C57Bl/6 control mice (black symbols) *versus Mtrr^gt/gt^* mice (grey symbols) at 7 weeks of age. Graphical data are presented as means ± SD. Blood from female (f, circles) and male (m, squares) mice was assessed. *n* = 3–5 mice per group. *A*, graphs indicating erythroid cell characteristics of C57Bl/6 and *Mtrr^gt/gt^* mice. MCV, mean corpuscular volume; RDW, red blood cell distribution width; RBC, red blood cells; Hct, haematocrit; Hb, haemoglobin; MCH, mean corpuscular haemoglobin; MCHC, mean corpuscular haemoglobin concentration; RPI, reticulocyte production index. *B*, representative peripheral blood smears from C57Bl/6 and *Mtrr^gt/gt^* mice. Blood from female (f) and male (m) mice was assessed. Black arrowheads indicate RBCs with Howell–Jolly bodies. White arrowhead indicates a microcytic cell. Scale bars: 10 μm. *C* **–** *E*, graphs indicating the percentage of RBCs that were microcytic (*C*), polychromatic (*D*) or contained Howell–Jolly bodies (HJBs, *E*) in peripheral blood smears of C57Bl/6 and *Mtrr^gt/gt^* mice. *F* **–** *L*, graphs indicating the number of platelets (*F*), neutrophils (*G*), white blood cells (WBC, *H*), lymphocytes (*I*), basophils (*J*), eosinophils (*K*) and monocytes (*L*) in peripheral blood of C57Bl/6 and *Mtrr^gt/gt^* mice. Statistical analysis: unpaired *t* tests were performed to independently compare C57Bl/6 females to *Mtrr^gt/gt^* females or C57Bl/6 males to *Mtrr^gt/gt^* males. ^*^ *P* < 0.05, ^**^ *P* < 0.01, ^***^ *P* < 0.001. [Color figure can be viewed at http://wileyonlinelibrary.com]

To determine whether the haematological effects of *Mtrr* deficiency were age related, the peripheral blood of C57Bl/6 and *Mtrr^gt/gt^* mice at 7 weeks of age was characterized. Blood profiles of young *Mtrr^gt/gt^* female and male mice were largely within the normal range compared to controls (Fig. 2). However, young male and female *Mtrr^gt/gt^* mice displayed significantly more microcytic RBCs than controls (*P* < 0.0001; Fig. 2 *B* and *C*), though the mean corpuscular volume and RBC distribution widths were unchanged (Fig. 2 *A*). Furthermore, significantly more microcytes were present in young *Mtrr^gt/gt^* mice than older *Mtrr^gt/gt^* mice (*P* = 0.008; Figs 1 *C* and 2 *C*). This difference was not present in young *versus* old C57Bl/6 mice (*P* > 0.50; Figs 1 *C* and 2 *C*). More Howell–Jolly bodies were also found in RBCs as early as 7 weeks in male and female *Mtrr^gt/gt^* mice compared to controls (Fig. 2 *B* and *E*). Altogether, these data suggest that the peripheral blood phenotype observed in *Mtrr^gt/gt^* mice largely emerges with age. However, the increased presence of Howell–Jolly bodies and microcytes precedes the appearance of the other blood phenotypes in mice with abnormal folate metabolism.

### Potential erythrocyte precursor differentiation defect in *Mtrr^gt/gt^* females

**Figure 3:**
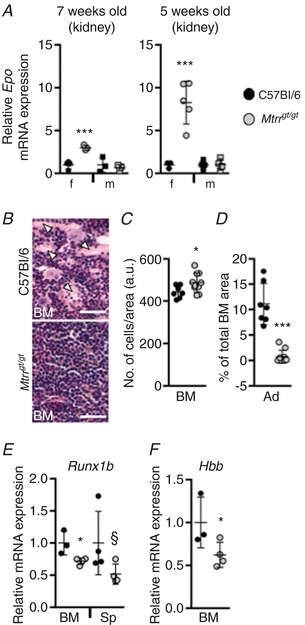
Abnormal erythroid differentiation, not haemolysis, is likely the cause of anaemia in *Mtrr^gt/gt^* female mice Graphical data show values from C57Bl/6 (black symbols) and *Mtrr^gt/gt^* (grey symbols) mice and are presented as means ± SD. *A*, RT‐qPCR analysis of erythropoietin (*Epo*) mRNA expression in kidneys from 7‐week‐old (young) and 5‐month‐old (older) C57Bl/6 and *Mtrr^gt/gt^* mice. mRNA expression levels of female (f, circles) and male (m, squares) mice are indicated. *n* = 3–5 mice were analysed per group. Data are presented as fold change compared to same sex C57Bl/6 controls (normalized to 1). *B*, representative images of H & E stained histological sections of bone marrow of older C57Bl/6 and *Mtrr^gt/gt^* female mice. White arrowheads indicate adipocytes. Scale bars: 10 μm. *C* and *D*, graphs indicating the number of bone marrow (BM) cells per designated area (a.u., arbitrary units, *C*) and percentage of total bone marrow area represented by adipocytes (Ad) in older C57Bl/6 and *Mtrr^gt/gt^* female mice (*D*). *E* and *F*, graphs showing an RT‐qPCR analysis of *Runx1b* in bone marrow (BM) and spleen (Sp) (*E*) and *Hbb* mRNA expression in bone marrow (*F*) of C57Bl/6 and *Mtrr^gt/gt^* older female mice. *n* = 3–4 mice were analysed per group. Data are presented as fold change compared to C57Bl/6 controls (normalized to 1). Statistical analysis: unpaired *t* tests were performed to independently compare C57Bl/6 females to *Mtrr^gt/gt^* females or C57Bl/6 males to *Mtrr^gt/gt^* males. ^§^ *P* = 0.05, ^*^ *P* < 0.05, ^***^ *P* < 0.001. [Color figure can be viewed at http://wileyonlinelibrary.com]

Erythropoietin (EPO) is a key hormone regulator of erythropoiesis. It is synthesized by the kidney to promote the formation of RBCs by acting on erythroid precursor cells in the bone marrow. Increased EPO levels are associated with anaemia (Koury & Ponka, 2004). As expected, *Mtrr^gt/gt^* male kidneys displayed normal levels of *Epo* mRNA expression (Fig. 3 *A*) compared to controls regardless of age. In contrast, a 3.0‐fold and 8.3‐fold increase in *Epo* mRNA levels were apparent in young and old *Mtrr^gt/gt^* female kidneys, respectively, compared to C57Bl/6 females (*P* < 0.001; Fig. 3 *A*). While anaemia was absent in young *Mtrr^gt/gt^* female mice (Fig. 2 *A*), increased *Epo* expression suggests that there may be an early, less severe anaemia phenotype that becomes progressively worse with age.

To further assess the effects of abnormal folate metabolism on haematopoiesis, histological sections of bone marrow of older females were analysed. *Mtrr^gt/gt^* females displayed hypercellular bone marrow compared to C57Bl/6 controls (Fig. 3 *B* and *C*). Additionally, the total area of bone marrow represented by adipocytes was substantially reduced in *Mtrr^gt/gt^* females (0.7% *versus* 11.1% in C57Bl/6 females; *P* = 0.0003) (Fig. 3 *B* and *D*). This observation may be important because marrow adipocytes are known to suppress the haematopoietic niche (Rosen *et al*. 2009). Furthermore, normal progression of erythroid differentiation depends upon reduced expression of *Runx1* (North *et al*. 2002), a gene that encodes a transcription factor normally expressed in haematopoietic stem cells in the bone marrow and colony forming units in the spleen (North *et al*. 2002; Maki *et al*. 2005). Hypercellular bone marrow and anaemia in mice are associated with decreased *Runx1* mRNA expression (Zhou *et al*. 2015). Indeed, *Runx1b* mRNA expression was significantly decreased in *Mtrr^gt/gt^* female bone marrow and spleens compared to controls (Fig. 3 *E*) as determined by quantitative reverse transcription PCR (RT‐qPCR) analysis. A downregulation of *Hbb* mRNA expression in bone marrow of *Mtrr^gt/gt^* females was also observed (Fig. 3 *F*). Under normal circumstances, the haemoglobin β chain complex (HBB) encourages erythrocyte maturation (Dore & Crispino, 2011). Altogether, these data support the hypothesis that the bone marrow of *Mtrr^gt/gt^* female mice displays an increased erythroid precursor population that may be unable to fully mature into erythrocytes causing anaemia.

**Figure 4:**
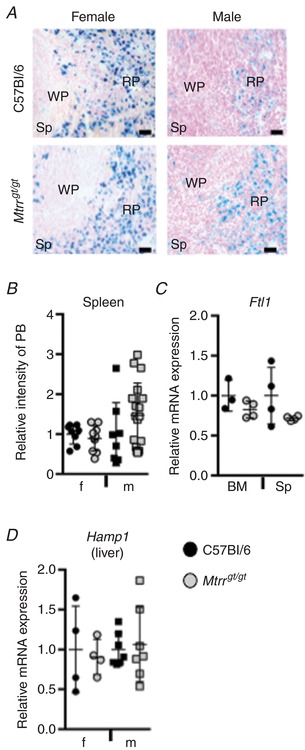
Haemolysis of erythrocytes is unlikely in *Mtrr^gt/gt^* mice *A*, representative histological sections of spleen from older C57Bl/6 and *Mtrr^gt/gt^* female (left‐hand panels) and male (right‐hand panels) mice. Sections were stained with Prussian blue to indicate iron deposits (blue). Nuclei, pink. WP, white pulp; RP, red pulp. Scale bars: 10 μm. *B*, the mean (±SD) relative intensity of Prussian blue staining in spleens of older C57Bl/6 (black symbols) and *Mtrr^gt/gt^* (grey symbols) females (f, circles) and males (m, squares) as determined by ImageJ software. *C*, RT‐qPCR analysis of *Ftl1* mRNA expression in bone marrow (BM) and spleens (Sp) of older C57Bl/6 (black) and *Mtrr^gt/gt^* (grey) female mice. *D*, RT‐qPCR analysis of *Hamp1* mRNA expression (gene that encodes hepcidin) in older C57Bl/6 (black) and *Mtrr^gt/gt^* (grey) livers. Female (f, circles) and male (m, squares) livers were assessed. For RT‐qPCR data: *n* = 3–8 mice were analysed per group and data are presented as fold change compared to C57Bl/6 controls (normalized to 1; mean ± SD). Statistical analysis: unpaired *t* tests were performed to independently compare C57Bl/6 females to *Mtrr^gt/gt^* females or C57Bl/6 males to *Mtrr^gt/gt^* males. [Color figure can be viewed at http://wileyonlinelibrary.com]

Instead of an erythroid differentiation defect in *Mtrr^gt/gt^* females, an alternative hypothesis proposes that RBC haemolysis is the cause of anaemia as was observed in folate‐deficient mice and *Slc46a1* knockouts (Bills *et al*. 1992; Salojin *et al*. 2011). Histological sections of C57Bl/6 and *Mtrr^gt/gt^* female and male spleens were exposed to Prussian blue stain to determine the degree of iron deposition as an indicator of haemolysis. No significant difference in iron deposition was observed in *Mtrr^gt/gt^* spleens compared to controls (Fig. 4 *A* and *B*). Next, using RT‐qPCR, we measured ferritin light chain 1 (*Ftl1*) mRNA expression since FTL1 mediates iron uptake and its expression is iron dependent (Arosio *et al*. 2015). *Ftl1* mRNA expression in bone marrow and spleens was within the normal range in *Mtrr^gt/gt^* compared to C57Bl/6 control females (*P* > 0.08; Fig. 4 *C*). Furthermore, we assessed hepatic *Hamp1* expression, a gene that encodes hepcidin, a hormone regulator of iron storage. Increased hepcidin production normally occurs when iron is abundant and is suppressed during erythropoiesis to make iron available for haemoglobin synthesis (Ganz & Nemeth, 2012). In *Mtrr^gt/gt^* females and males, *Hamp1* mRNA expression was similar to C57Bl/6 controls (Fig. 4 *D*). Together, these data suggest that anaemia caused by *Mtrr* deficiency in female mice is unlikely to be due to haemolysis of RBCs.

### Splenomegaly and extramedullary erythropoiesis observed in female *Mtrr^gt/gt^* mice

**Figure 5:**
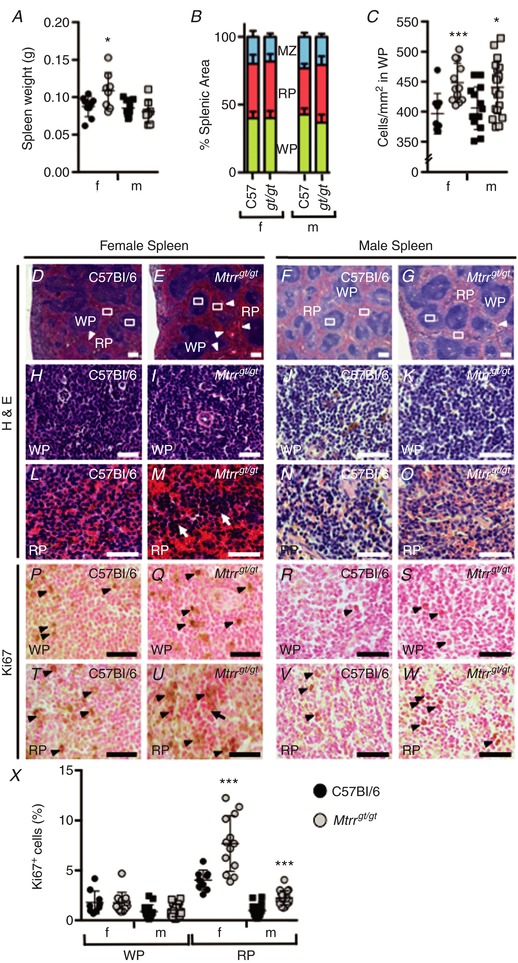
*Mtrr^gt/gt^* female mice display splenomegaly and potential extramedullary haematopoiesis Graphical data show values from older C57Bl/6 (black symbols) and *Mtrr^gt/gt^* (grey symbols) mice and are presented as means ± SD. Histological sections show representative spleens from older C57Bl/6 and *Mtrr^gt/gt^* female mice. *A*, spleen weights in C57Bl/6 and *Mtrr^gt/gt^* mice. Weights of female (f, circles) and male (m, squares) mice are shown. *B*, graph indicating the percentage of splenic area represented by the major structural components including white pulp (WP; green bar), red pulp (RP; pink bar) and marginal zone (MZ; blue bar) in C57Bl/6 (C57) and *Mtrr^gt/gt^* (*gt/gt*) female (f) and male (m) spleens. *C*, graph quantifying white pulp (WP) cell density in the spleens of older C57Bl/6 and *Mtrr^gt/gt^* female (f, circles) and male (m, squares) mice. *D* **–** *O*, representative histological sections of spleens stained with H & E. Female and male spleens are shown from older C57Bl/6 and *Mtrr^gt/gt^* mice. White boxes in *D* **–** *G* indicate regions of higher magnification in images directly below including white pulp (WP, *H* **–** *K*) and red pulp (RP, *L* **‐** *O*). White arrowheads indicate regions of fibrosis. Arrows in *M* indicate regions of possible extramedullary haematopoiesis. Scale bars: *D* **–** *G*, 200 μm; *H* **–** *O*, 10 μm. *P* **–** *W*, histological sections of spleens that were immunostained for the mitosis marker Ki67 (brown, arrowheads). Nuclei, pink. Female and male spleens from older C57Bl/6 and *Mtrr^gt/gt^* mice are represented. *P* **–** *S*, white pulp (WP). *T* **–** *W*, red pulp (RP). Arrow in *U* indicates region of possible extramedullary haematopoiesis. Scale bars: 10 μm. *X*, the percentage of Ki67‐positive cells in white pulp (WP) and red pulp (RP) of histological sections of spleens from C57Bl/6 (black symbols) and *Mtrr^gt/gt^* (grey symbols) mice. Data from female (f, circles) and male (m, squares) spleens are represented as means ± SD. Statistical analysis: unpaired *t* tests were performed to independently compare C57Bl/6 females to *Mtrr^gt/gt^* females or C57Bl/6 males to *Mtrr^gt/gt^* males. ^*^ *P* < 0.05, ^***^ *P* < 0.005. [Color figure can be viewed at http://wileyonlinelibrary.com]

Splenomegaly and extramedullary haematopoiesis are often associated with anaemia in humans. Older *Mtrr^gt/gt^* female mice, which were anaemic (Fig. 1 *A*), displayed splenomegaly since the average splenic weight was significantly higher than C57Bl/6 female spleens (*P* < 0.05; Fig. 5 *A*). A similar effect was absent in *Mtrr^gt/gt^* males (Fig. 5 *A*), which did not display anaemia in peripheral blood (Fig. 1 *A*). Further analysis of splenic regions revealed that the white pulp, red pulp and marginal zone displayed a similar regional area (Fig. 5 *B*) and organization (Fig. 5 *D*–*G*) in *Mtrr^gt/gt^* female and male spleens compared to C57Bl/6 controls. However, a high degree of fibrosis was apparent in female *Mtrr^gt/gt^* spleens (Fig. 5 *D* and *E*), which may indicate degenerative lesions and disease (Cesta, 2006). Cell counts in histological sections revealed an increased density of cells in the white pulp of *Mtrr^gt/gt^* female and male spleens (Fig. 5 *C*, *H*–*K*), though there was no change in occurrence of mitotic cells in this region as indicated by Ki67 immunostaining (Fig. 5 *P*–*S* and *X*). Although difficult to quantify, histological sections of red pulp in *Mtrr^gt/gt^* female spleens were qualitatively more densely packed with mature erythrocytes (Fig. 5 *L* and *M*). Clusters of cells, possibly erythroid progenitors, were apparent in the red pulp of *Mtrr^gt/gt^* female spleens (Fig. 5 *M*), though not in *Mtrr^gt/gt^* male spleens (Fig. 5 *O*) or controls (Fig. 5 *L* and *N*). This observation may indicate the presence of extramedullary erythropoiesis, which is consistent with anaemia detected in peripheral blood (Cesta, 2006). Furthermore, this extramedullary haematopoiesis was associated with a decrease in *Runx1b* mRNA expression in *Mtrr^gt/gt^* female spleens (*P* = 0.05; Fig. 3 *E*) and an overall increase in Ki67‐expressing cells in the red pulp of *Mtrr^gt/gt^* spleens including in regions of presumed extramedullary haematopoiesis in *Mtrr^gt/gt^* females (Fig. 5 *T*–*X*). Overall, these data suggest that abnormal folate metabolism results in female‐specific macrocytic anaemia associated with splenomegaly and extramedullary erythropoiesis.

### Presence of a parental *Mtrr^gt^* allele leads to abnormal haematological profiles in their wildtype daughters

**Figure 6:**
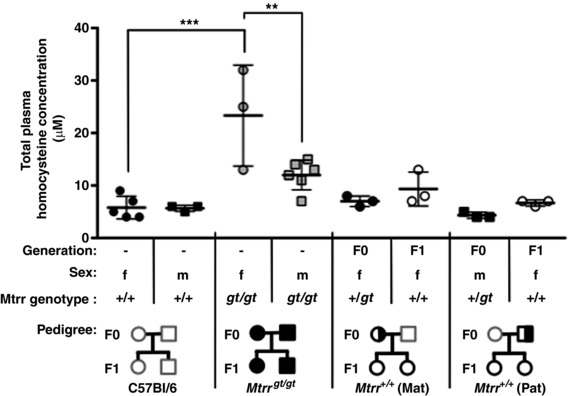
Parental *Mtrr^gt^* heterozygosity does not alter folate metabolism in their wildtype daughters Graph shows total homocysteine concentrations (means ± SD) measured by LC‐MS/MS in plasma of female (f, circles) and male (m, squares) mice from the designated genotype and pedigree. Values from female and male C57Bl/6 (control), *Mtrr^+/gt^* and *Mtrr^gt/gt^* mice were previously published (Padmanabhan *et al*. 2013). Total homocysteine concentrations in the plasma of wildtype (*Mtrr^+/+^*) female mice (i.e. the F1 generation) derived from an *Mtrr^+/gt^* mother (Mat) or *Mtrr^+/gt^* father (Pat) (i.e. the F0 generation) are indicated. *n* = 3–6 mice per group were assessed. Statistical analysis: a one‐way ANOVA with Tukey multiple comparison tests was performed. ^**^ *P* < 0.01, ^***^ *P* < 0.005. Pedigree legend: circle, female; square, male; grey outline, C57Bl/6 mice; black outline, *Mtrr* mouse line; white fill, *Mtrr^+/+^*; half white/half black fill, *Mtrr^+/gt^*; black fill, *Mtrr^gt/gt^*.

We previously showed that when mice are carriers of an *Mtrr^gt^* allele, it is sufficient to cause abnormal phenotypes several wildtype generations later (Padmanabhan *et al*. 2013). This multigenerational effect occurs only through the maternal lineage (i.e. the maternal grandmother or maternal grandfather) (Padmanabhan *et al*. 2013). Therefore, to explore whether there is a generational effect of abnormal folate metabolism on haematopoiesis, we completed a similar analysis as above in wildtype F1 females of an *Mtrr^+/gt^* parent. To do this, *Mtrr^+/gt^* male mice were mated with C57Bl/6 females and *Mtrr^+/gt^* female mice were mated with C57Bl/6 males to assess a paternal and maternal effect, respectively. All analyses were completed in non‐pregnant F1 females and C57Bl/6 female mice were used as controls. First, using tandem mass spectrometry, we determined that the wildtype F1 females from each pedigree showed total plasma homocysteine concentrations that were within the normal range of C57Bl/6 female mice (Fig. 6). This result indicates that one‐carbon metabolism in wildtype F1 females remains unaffected by parental *Mtrr^gt^* heterozygosity.

**Figure 7:**
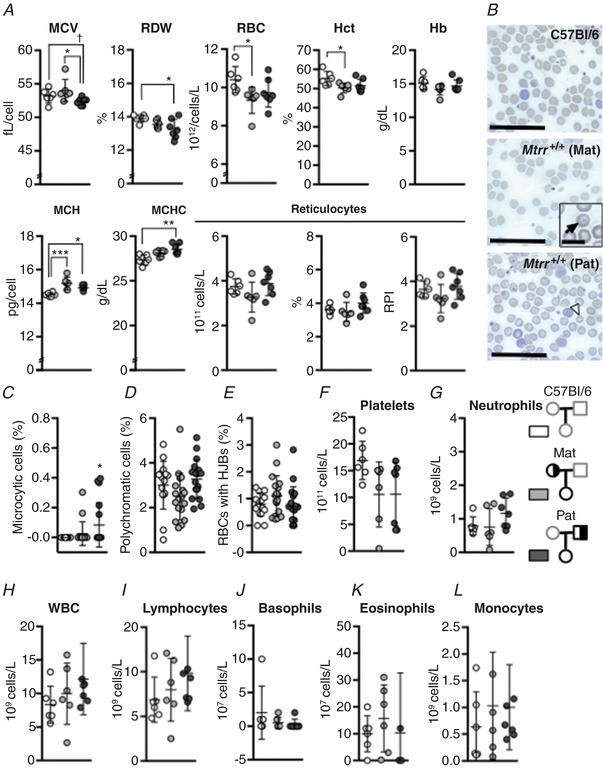
*Mtrr^gt^* heterozygosity causes haematological defects in peripheral blood profiles of their adult wildtype daughters in a parent‐specific manner Haematological profiles of peripheral blood from C57Bl/6 control female mice (white) *versus* wildtype (*Mtrr^+/+^*) F1 female mice derived from either an *Mtrr^+/gt^* mother (Mat; light grey) or an *Mtrr^+/gt^* father (Pat; dark grey). Graphical data are presented as means ± SD. *n* = 6–7 mice were analysed per group. *A*, graphs indicating erythroid cell characteristics of C57Bl/6 females and wildtype F1 females derived from either an *Mtrr^+/gt^* mother or father. MCV, mean corpuscular volume; RDW, red blood cell distribution width; RBC, red blood cells; Hct, haematocrit; Hb, haemoglobin; MCH, mean corpuscular haemoglobin; MCHC, mean corpuscular haemoglobin concentration; RPI, reticulocyte production index. *B*, representative images of peripheral blood smears from C57Bl/6 females and wildtype F1 females derived from an *Mtrr^+/gt^* mother (Mat) or father (Pat). Inset: black arrow indicates a codocyte. White arrowhead indicates a microcytic cell. Scale bars: 10 μm; inset, 2.5 μm. *C* **–** *E*, graphs indicating the percentage of RBCs that were microcytic (*C*), polychromatic (*D*) or contained Howell–Jolly bodies (HJBs, *E*) in peripheral blood smears of C57Bl/6 females and wildtype F1 females derived from an *Mtrr^+/gt^* mother or father. *F* **–** *L*, graphs indicating the number of platelets (*F*), neutrophils (*G*), white blood cells (WBC, *H*), lymphocytes (*I*), basophils (*J*), eosinophils (*K*) and monocytes (*L*) in the peripheral blood of C57Bl/6 females and wildtype F1 females derived from an *Mtrr^+/gt^* mother or father. Statistical analysis: ordinary one‐way ANOVA with Tukey multiple comparison tests was performed. ^†^ *P* = 0.060, ^*^ *P* < 0.05, ^**^ *P* < 0.005, ^***^ *P* < 0.001. Pedigree legend: circle, female; square, male; grey outline, C57Bl/6 mice; black outline, *Mtrr* mouse line; white fill, *Mtrr^+/+^*; half white/half black fill, *Mtrr^+/gt^*. [Color figure can be viewed at http://wileyonlinelibrary.com]

Next, peripheral blood was collected from 4‐ to 5‐month‐old wildtype daughters (*n* = 6–7 mice) from each pedigree and compared to blood from similar aged C57Bl/6 control females (*n* = 6 mice). Comparable to *Mtrr^gt/gt^* female mice, the peripheral blood of wildtype F1 females derived from *Mtrr^+/gt^* mothers exhibited reduced RBC counts and haemocrit compared to controls (Fig. 7 *A*) indicating anaemia. However, these cells were not macrocytic as determined by mean corpuscular volume (Fig. 7 *A*). On the other hand, wildtype F1 females derived from *Mtrr^+/gt^* fathers did not display anaemia since their peripheral RBC counts were within the normal range (Fig. 7 *A*). Also in contrast to the effects of *Mtrr^gt^* homozygosity in females and of maternal *Mtrr^gt^* heterozygosity, the RBCs of these wildtype F1 females showed a decreasing trend in mean corpuscular volume compared to C57Bl/6 controls (*P* = 0.060, Fig. 7 *A*) and were less variable in size compared to C57Bl/6 controls as demonstrated by a lower RBC distribution width (*P* = 0.04; Fig. 7 *A* and *B*). In support of this finding, significantly more microcytic RBCs were present in the blood smears of F1 females derived from *Mtrr^+/gt^* fathers compared to controls (Fig. 7 *B* and *C*), an observation comparable to *Mtrr^gt/gt^* males (Fig. 1 *C*).

**Figure 8:**
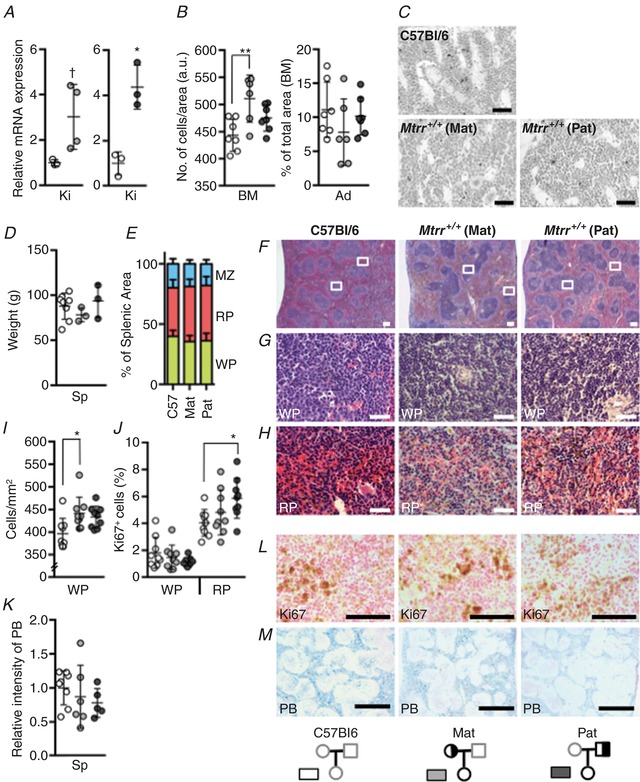
Wildtype F1 females derived from an *Mtrr^+/gt^* parent display hypercellular bone marrow and no splenic defects Graphical data show values from C57Bl/6 (white) and wildtype F1 females derived from an *Mtrr^+/gt^* mother (Mat; light grey) or *Mtrr^+/gt^* father (Pat; dark grey) mice. Values are presented as means ± SD. *n* = 3–5 mice per group were assessed. *A*, RT**‐**qPCR analysis of erythropoietin (*Epo*) mRNA expression in kidneys (Ki). Data are presented as fold difference compared to C57Bl/6 controls (normalized to 1). *B*, graphs indicating the number of bone marrow (BM) cells per designated area (a.u., arbitrary units; left‐hand graph) and the percentage of total bone marrow area represented by adipocytes (Ad; right‐hand graph). *C*, histological sections of bone marrow from C57Bl/6 and wildtype F1 females derived from *Mtrr^+/gt^* mothers (Mat) or *Mtrr^+/gt^* fathers (Pat). Scale bars: 10 μm. *D*, the average splenic (Sp) weight for each group is assessed. *E*, data indicating the percentage of splenic area divided into major components including white pulp (WP, green bar), red pulp (RP, pink bar) and marginal zone (MZ, blue bar) for each female group. *F* **–** *H*, H & E‐stained histological sections of spleens from C57Bl/6 females (left panel), and wildtype F1 females derived from an *Mtrr^+/gt^* mother (Mat; centre panel) or an *Mtrr^+/gt^* father (Pat; right panel). White boxes in *F* indicate regions of higher magnification shown in panels directly below and show the white pulp (WP, *G*) or red pulp (RP, *H*) from each pedigree. Scale bars: *F*, 200 μm; *G* **–** *H*, 10 μm. *I* **–** *J*, graphs showing the number of splenic white pulp (WP) cells in a defined area (mm^2^, *I*) and the percentage of Ki67‐positive cells in the white pulp (WP) and red pulp (RP, *J*) of spleens from each female group. *K*, the relative intensity of Prussian blue (PB) stain in spleens to indicate iron deposition in each female group as determined by ImageJ software. *L*, histological sections of splenic red pulp from C57Bl/6 females and wildtype F1 females derived from *Mtrr^+/gt^* mothers (Mat) or fathers (Pat) that were immunostained for Ki67 (brown). Nuclei, pink. Scale bar: 10 μm. *M*, histological sections of spleens from each female group stained with Prussian blue (PB) to indicate iron deposition (blue). Nuclei, pink. Scale bar: 100 μm. Statistical analysis: unpaired *t* tests were performed to independently compare F1 *Mtrr^+/+^* females to the respective C57Bl/6 female controls (*A*); ordinary one‐way ANOVA with multiple comparison tests was performed to compare wildtype F1 females to the C57Bl/6 control (*B*, *D* and *E*, *I*–*K*). ^†^ *P* = 0.066, ^*^ *P* < 0.05, ^**^ *P* < 0.01. Pedigree legend: circle, female; square, male; grey outline, C57Bl/6 mice; black outline, *Mtrr* mouse line; white fill, *Mtrr^+/+^*; half white/half black fill, *Mtrr^+/gt^*. [Color figure can be viewed at http://wileyonlinelibrary.com]

Even though maternal *Mtrr^gt^* heterozygosity caused anaemia in their wildtype daughters and paternal *Mtrr^gt^* heterozygosity did not, reticulocyte counts and reticulocyte production indices were comparable to controls in both cases (Fig. 7 *A* and *D*). Despite this observation, renal *Epo* mRNA expression was increased in wildtype F1 females from both pedigrees: a 3.0‐fold increase caused by *Mtrr^+/gt^* mothers (*P* = 0.066) and a 4.4‐fold increase caused by *Mtrr^+/gt^* fathers (*P* = 0.03) relative to controls (Fig. 8 *A*). The former result was not statistically significant. However, hypercellular bone marrow was observed only in wildtype F1 females from an *Mtrr^+/gt^* mother (*P* = 0.006; Fig. 8 *B* and *C*), likely in response to anaemia and increased *Epo* expression. This result suggests that the haematopoietic stem cells of F1 females might be more sensitive to maternal rather than paternal heterozygosity. Yet in contrast to older *Mtrr^gt/gt^* females (Fig. 1 *A* and *D*), reticulocyte counts and reticulocyte production indices were unaltered by a parental effect (Fig. 7 *A* and *D*). This may indicate that the effects of parental *Mtrr^gt^* heterozygosity are less severe than intrinsic *Mtrr* deficiency.

The WBC population and myeloid lineages beyond erythrocytes were not significantly affected in F1 females by an *Mtrr^+/gt^* parental genotype (Fig. 7 *F*–*L*). Although there was no significant increase in the number of Howell–Jolly bodies present in the erythrocytes of the wildtype F1 females regardless of pedigree (Fig. 7 *E*), maternal heterozygosity was associated with the appearance of codocytes (target cells) in at least one of the wildtype F1 females assessed (Fig. 7 *B*). Codocytes were not apparent in *Mtrr^gt/gt^* mice or wildtype F1 females derived from *Mtrr^+/gt^* fathers (Figs 1 *B*, 2 *B* and 7 *B*), and are often indicative of liver disease, iron deficiency, or splenic defects (Mehta, 2007). Altogether, these data reveal a parent‐specific effect of abnormal folate metabolism such that wildtype females of *Mtrr^+/gt^* mothers displayed normocytic anaemia and wildtype females of *Mtrr^+/gt^* fathers exhibited RBC microcytosis.

### A parental effect of the *Mtrr^gt^* allele on F1 female spleen histology is not evident

Unlike *Mtrr^gt/gt^* females, spleen weight and gross histology was normal in wildtype F1 females from either pedigree compared to C57Bl/6 controls (Fig. 8 *D*–*I*) with the exception of the white pulp cell density, which was slightly increased in F1 females derived from an *Mtrr^+/gt^* mother (*P* = 0.01; Fig. 8 *I*). Despite this, Ki67^+^ cells appeared at a normal frequency in the white and red pulp of these F1 females compared to controls (Fig. 8 *J* and *L*). However, paternal *Mtrr^gt^* heterozygosity was associated with a slightly higher percentage of mitotic cells in the red pulp of spleens from wildtype F1 females (*P* = 0.04; Fig. 8 *J* and *L*) yet no clear regions of extramedullary haematopoiesis were observed. Furthermore, the spleens of F1 females from both pedigrees displayed normal iron deposition (Fig. 8 *K* and *M*) suggesting an absence of excessive haemolysis. Altogether, these data indicate that parental *Mtrr^gt^* heterozygosity does not lead to overt splenic defects including extramedullary erythropoiesis or fibrosis.

## Discussion

We have shown here that abnormal metabolism of folate caused by the *Mtrr^gt^* mutation in mice results in haematopoietic phenotypes that are sex and age dependent. More specifically, *Mtrr^gt/gt^* female mice displayed macrocytic anaemia, which may be due to defective erythroid differentiation at the exclusion of haemolysis. This was accompanied by robust induction of renal *Epo* mRNA expression, hypercellular bone marrow and extramedullary haematopoiesis. In contrast, the male response to abnormal folate metabolism differed since *Mtrr^gt/gt^* male mice were not anaemic but did display erythrocytic macrocytosis and lymphopenia. Regardless of sex, these blood phenotypes developed with age, as the haematopoietic profiles of young *Mtrr^gt/gt^* mice were largely normal though subtle erythrocyte abnormalities hint at an early, milder defect. Remarkably, we also showed that when a parent is a carrier of the *Mtrr^gt^* allele, it resulted in haematological defects in their wildtype adult daughters. However, the type of defect was dependent upon the sex of the parent. Maternal *Mtrr^gt^* heterozygosity resulted in normocytic anaemia with increased renal *Epo* and hypercellular bone marrow in wildtype daughters. In this case, it was unclear whether erythrocytic differentiation or longevity was affected. In contrast, paternal *Mtrr^gt^* heterozygosity caused erythrocytic microcytosis not associated with anaemia despite increased renal *Epo* mRNA expression in wildtype daughters. Altogether, our data show that abnormal folate metabolism affects haematopoiesis in an age‐, sex‐ and parent‐of‐origin‐specific manner.

Humans that are folate deficient or carry mutations in genes encoding for folate uptake (e.g. *SLC46A1*) or metabolism (e.g. *MTR*, *MTRR*) exhibit megaloblastic anaemia, a type of macrocytic anaemia (Gulati *et al*. 1996; Leclerc *et al*. 1996; Zavadakova *et al*. 2002; Vilaseca *et al*. 2003). An important diagnostic criterion for megaloblastic anaemia is the presence of neutrophils with hyper‐segmented nuclei (Doig, 2007). While mature neutrophils in mice are fully segmented, natural twisting and folding of their nuclei makes it difficult to distinguish folds from segments (Zhou *et al*. 2015). *Slc46a1^−/−^* mice were found to have macrocytic anaemia with multi‐lobed polymorphonuclear neutrophils (Salojin *et al*. 2011). Even though *Mtrr^gt/gt^* female mice display macrocytic anaemia, we were unable to determine whether the anaemia fulfils megaloblastic status. Furthermore, our finding that older *Mtrr^gt/gt^* female mice have increased reticulocyte counts contradicts most human cases of megaloblastic anaemia associated with folate deficiency wherein reticulocyte numbers are usually low (Koury & Ponka, 2004). The difference might be explained by type of insult or the species assessed. Regardless, the *Mtrr^gt^* mouse line is a novel model to assess the role of folate metabolism in anaemia.

Clear similarities exist between the haematological phenotypes of known mouse models of folate deficiency/defective folate uptake (Bills *et al*. 1992; Koury *et al*. 1997; Zhao *et al*. 2001; Salojin *et al*. 2011) and abnormal folate metabolism in the *Mtrr^gt^* model (this study). All models display anaemia associated with increased erythropoietin expression, increased erythrocytic progenitors, and splenomegaly associated with extramedullary haematopoiesis. Hypercellular bone marrow was only observed in *Mtrr^gt/gt^* mice (this study) and *Slc46a1^−/−^* mice (Salojin *et al*. 2011). In folate‐deficient mice or in *Slc46a1* mutants, erythropoiesis fails during the maturation phase and anaemia may be due to increased haemolysis of differentiating erythrocytes (Koury *et al*. 1997; Salojin *et al*. 2011). This differs with the *Mtrr^gt^* model where erythrocyte differentiation and/or maturation were abnormal, yet erythroid apoptosis as a contributor to anaemia was unlikely since iron deposition and *Hamp1* mRNA expression were normal. This inconsistency may reflect a difference in methodology or in the severity of the initiating defect. For example, the *Slc46a1* mutation prevents the uptake of folate (Salojin *et al*. 2011) akin to dietary folate deficiency. In *Mtrr^gt/gt^* mutants, folate is transported into the cells as usual but its metabolism is abnormal (Elmore *et al*. 2007). Furthermore, the *Mtrr^gt^* allele is a hypomorphic mutation and has a knockdown effect such that in *Mtrr^gt/gt^* mutants, a wildtype *Mtrr* transcript is weakly expressed allowing one‐carbon metabolism to progress albeit in a significantly reduced capacity (Elmore *et al*. 2007). Therefore, the haematological phenotype in *Mtrr^gt/gt^* mice is different from and/or not as severe as that in *Slc46a1^−/−^* mice.

The onset of haematopoietic defects associated with abnormal folate uptake or metabolism varies between models and also likely depends upon the severity of the disruption. For instance, mouse embryos lacking SLC19A1, another protein that mediates folate uptake (also known as RFC1), die at mid‐gestation with a discernable absence of erythropoiesis (Gelineau‐van Waes *et al*. 2008). When rescued from embryonic lethality by maternal folic acid supplementation, *Slc19a1^−/−^* mutants die shortly after birth due to an absence of haematopoiesis in the bone marrow, spleen and liver (Zhao *et al*. 2001), which might implicate regulatory defects in the placenta and fetal liver (Mikkola *et al*. 2005). Although the timing of phenotypic onset is unknown, *Slc46a1^−/−^* mice display severe macrocytic anaemia by 4–6 weeks (Salojin *et al*. 2011). On the other hand, *Mtrr^gt/gt^* female mice do not display macrocytic anaemia until a later stage. Yet, as early as 7 weeks of age, *Mtrr^gt/gt^* females exhibit a milder phenotype including increased renal *Epo* mRNA expression and erythrocytic defects (e.g. more Howell–Jolly bodies). This suggests that blood health decreases with age in response to *Mtrr* deficiency. Overall, folate metabolism appears to play a role in embryonic and adult haematopoiesis.

It was hypothesized that folate‐deficient erythroid progenitors are unable to withstand the rapid proliferation that occurs during their formation and, ultimately, undergo apoptosis leading to anaemia (Koury *et al*. 1997). Anaemia associated with folate deficiency was previously attributed to a lack of thymidylate synthesis resulting in uracil misincorporation into DNA and increased DNA breaks (Wickramasinghe & Fida, 1994; Blount *et al*. 1997). Others have shown that folate levels and DNA uracil content do not correlate in RBCs of folate‐deficient mice, though increased micronuclei formation was detected indicating the presence of chromosomal damage (Swayne *et al*. 2012). Female and male *Mtrr^gt/gt^* mice at both ages assessed had a high frequency of Howell–Jolly bodies (i.e. DNA remnants). Whether these bodies indicate chromosomal instability or an inability to undergo nuclear extrusion during the maturation in erythroid precursor cells is unclear. Certainly, other periods of rapid proliferation, such as fetoplacental development, require folate since embryonic lethality occurs with varying degrees of penetrance in mouse models of defective folate uptake or metabolism (Piedrahita *et al*. 1999; Swanson *et al*. 2001; Gelineau‐van Waes *et al*. 2008; Padmanabhan *et al*. 2013). Whether uracil misincorporation and/or genetic instability are the primary cause of developmental phenotypes has not yet been pursued in these models.

An alternative hypothesis to genetic instability as a cause for macrocytic anaemia in *Mtrr^gt/gt^* female mice is epigenetic instability. DNA methylation patterns are dynamic during haematopoietic stem cell commitment and differentiation into the erythroid lineage (Madzo *et al*. 2014). Particularly, the binding sites of GATA2 and RUNX1 among other transcription factors that are known regulators of erythroid differentiation gain 5‐hydroxymethylcytosine as differentiation occurs (Madzo *et al*. 2014). Interestingly, blood phenotypes occur in a number of mouse models with defective regulation of DNA methylation (Tefferi *et al*. 2009; Ko *et al*. 2010; Sasaki *et al*. 2012; Russler‐Germain *et al*. 2014; Kunimoto *et al*. 2017). For example, a loss‐of‐function mutation in the mouse gene *Aid*, which encodes a critical enzyme involved in active DNA demethylation, results in DNA hypermethylation, an expansion of myeloid cells, and reduced erythroid progenitors resulting in anaemia (Kunimoto *et al*. 2017). Importantly, folate metabolism is required for the transmission of one‐carbon methyl groups for cellular methylation (Jacob *et al*. 1998; Ghandour *et al*. 2002) including DNA and histone methylation. Global DNA hypomethylation and locus‐specific dysregulation of DNA methylation in association with the misexpression of genes occurs in *Mtrr^gt/gt^* tissue (Padmanabhan *et al*. 2013). Therefore, it is possible that a similar dysregulation of DNA methylation occurs in the erythroid lineage affecting its differentiation, maturation and/or potential for survival. The effects of epigenetic instability caused by abnormal folate metabolism on haematopoiesis should be pursued in future studies. Notably, genetic instability and epigenetic instability due to folate deficiency or abnormal folate metabolism need not be mutually exclusive mechanisms.

Prior to this study, there was no clear evidence of a sexually dimorphic response to folate deficiency in humans or mice with respect to anaemia. While only folate‐deficient female mice have been assessed for haematological effects (Bills *et al*. 1992; Koury *et al*. 1997), the phenotypic data from *Slc46a1^−/−^* female and male mice was pooled (Salojin *et al*. 2011). We showed here that even though both female and male *Mtrr^gt/gt^* mice exhibit similar haematological phenotypes (e.g. macrocytic erythrocytes, more Howell–Jolly bodies), their phenotypes at large were unequal since only *Mtrr^gt/gt^* females exhibited anaemia associated with increased *Epo* mRNA expression, splenomegaly and extramedullary haematopoiesis, and only *Mtrr^gt/gt^* males exhibited lymphopenia. Apart from blood, only two other sex‐specific adult phenotypes have been identified in *Mtrr^gt/gt^* mice (Elmore *et al*. 2007; Padmanabhan *et al*. 2013). Male *Mtrr^gt/gt^* mice display lower plasma total homocysteine levels than *Mtrr^gt/gt^* females (Padmanabhan *et al*. 2013). Whether the concentration of plasma total hyperhomocysteine influences the haematopoietic profile in *Mtrr^gt/gt^* mice in a sexually dimorphic manner, if at all, is unknown. Additionally, adult *Mtrr^gt/gt^* males showed reduced weight gain over time compared to wildtype littermates, a phenotype not observed in *Mtrr^gt/gt^* females (Elmore *et al*. 2007). This, together with the observations in this study, implies that some metabolic and physiological responses to *Mtrr* deficiency are sex specific.

The appearance of anaemia in *Mtrr^gt/gt^* female mice and not males might be attributed to hormonal or epigenetic differences. For instance, the self‐renewal rate of haematopoietic stem cells is higher in females and in the presence of oestrogen (Nakada *et al*. 2014). Oestrogen receptor expression is reduced during folate deficiency in male and female mice (Gao *et al*. 2012; Yuan *et al*. 2017). Whilst not yet explored in *Mtrr^gt/gt^* mice, it is plausible that *Mtrr^gt/gt^* females might be more sensitive to the physiological effects of reduced oestrogen signalling resulting in ineffective haematopoiesis. Alternatively, since folate metabolism promotes methyl group availability, defective folate metabolism may differentially affect the maintenance of DNA methylation patterns in males and females leading to the dysregulation of gene expression in a sexually dimorphic manner. While sexual dimorphism of developmental phenotypes in *Mtrr^gt/gt^* conceptuses at mid‐gestation was not apparent shortly after DNA methylation patterns are established (Iurlaro *et al*. 2017; Padmanabhan *et al*. 2017), it is still possible that DNA methylation profiles are unequally maintained between female and male *Mtrr^gt/gt^* conceptuses or adults. Maternal folate supplementation results in the differential methylation of DNA in female and male offspring (Penailillo *et al*. 2015; Barua *et al*. 2016; Qian *et al*. 2016; Caffrey *et al*. 2018), which implies that intrinsic abnormal folate metabolism may lead to a similar deregulatory effect. Whether sex‐specific epigenetic dysregulation in the haematopoietic lineage of the *Mtrr^gt/gt^* model results in anaemia is yet to be determined and will require an unbiased whole methylome approach.

It is widely known that maternal folate deficiency increases disease/phenotype risk in their offspring, with neural tube defects as the most publicized example (MRC Vitamin Study Research Group, 1991). Paternal folate deficiency also causes craniofacial and musculoskeletal malformations (Lambrot *et al*. 2013). Nevertheless, little focus has been placed on the parental effects of abnormal folate uptake or metabolism on adult‐onset disease in their offspring. Our study supports the hypothesis that when either parent is a carrier of the *Mtrr^gt^* allele, it is sufficient to cause blood defects in their adult wildtype daughters. Phenotypic similarities and differences exist between intrinsic (e.g. *Mtrr^gt/gt^* mice) and parental (e.g. wildtype F1 females of an *Mtrr^+/gt^* parent) abnormal folate metabolism. For example, both *Mtrr^gt/gt^* females and wildtype F1 females derived from an *Mtrr^+/gt^* mother displayed anaemia associated with increased renal *Epo* and hypercellular bone marrow. However, *Mtrr^gt/gt^* females had splenomegaly, erythrocytes that were macrocytic, and increased reticulocyte counts, all parameters of which were normal in wildtype F1 females from *Mtrr^+/gt^* mothers. This suggests that the effects of intrinsic *Mtrr* deficiency are more severe than maternal *Mtrr^gt^* heterozygosity. In contrast, the wildtype daughters of *Mtrr^+/gt^* males showed opposing phenotypes to *Mtrr^gt/gt^* males including erythrocytic microcytosis *versus* macrocytosis, increased renal *Epo* mRNA levels *versus* normal levels, and a trend towards lymphocytosis *versus* lymphopenia, respectively. The above compares the haematological effects of parental *Mtrr^+/gt^* heterozygosity with intrinsic *Mtrr^gt/gt^* homozygosity. While mice with *Mtrr^+/gt^* and *Mtrr^gt/gt^* genotypes are not metabolically the same (Elmore *et al*. 2007; Padmanabhan *et al*. 2013), it is not possible to assess the offspring of *Mtrr^gt/gt^* parents without the potential compounding effect of the offspring's *Mtrr* genotype. Yet, it is remarkable that one parental *Mtrr^gt^* allele is sufficient to cause haematological effects in wildtype offspring.

The molecular mechanism behind the parental inheritance of blood phenotypes in the *Mtrr^gt^* mouse model is not well understood and likely differs to some extent depending on which parent is the *Mtrr^gt^* heterozygote. Folate metabolism is normal in the wildtype F1 generation as determined by normal plasma total homocysteine levels indicating that the metabolic defect lies solely with the parent. Abnormal folate metabolism may lead to the inheritance of *de novo* DNA mutations and/or defective epigenetic factors (e.g. DNA methylation, protamine/histone methylation, non‐coding RNAs) with the potential to alter gene expression and cause an adult phenotype(s) in their wildtype offspring, as others have described in non‐folate‐related models (Blake & Watson, 2016; Chen *et al*. 2016; Huypens *et al*. 2016). Whole genome, methylome and transcriptome studies in the bone marrow of *Mtrr^+/gt^* parents and their wildtype daughters will reveal inherited *de novo* genetic mutations or epigenetic targets that cause anaemia or macrocytosis. A maternal effect of the *Mtrr^gt^* mutation also implicates an atypical uterine environment during fetal development of the wildtype F1 generation or abnormal milk content. The nature of the epigenetic and physiological abnormalities in pregnant *Mtrr^+/gt^* females that have the potential to cause late‐onset haematological defects in the adult F1 generation are yet to be explored, but anaemia during pregnancy and lactation, itself, might play a role. Beyond this study, it will become important to explore parental folate status or parental mutations in folate‐related genes in patients to determine the cause of unexplained anaemia.

## Additional information

### Competing interests

None declared.

### Author contributions

This work was performed in the Department of Physiology, Development and Neuroscience at the University of Cambridge located in Cambridge, UK. All authors designed and conducted the research, analysed the data, and wrote and edited the manuscript. E.D.W. has primary responsibility for content. All authors read and approved the final manuscript and agree to be accountable for all aspects of the work. All persons designated as authors qualify for authorship, and all those who qualify for authorship are listed.

### Funding

The work was funded by the Lister Institute for Preventative Medicine, a Next Generation Fellowship from the Centre for Trophoblast Research, the Returning Carer's grant scheme (Cambridge) and an Isaac Newton Trust/Wellcome Trust ISSF/University of Cambridge joint research grant (to E.D.W.). The following financial support was given: Centre for Trophoblast Research Graduate Studentship (to N.P.), Newnham College (Cambridge) studentship and A.G. Leventis scholarship (to K.M.), H.E. Durham Fund grant and Downing College (Cambridge) summer studentship (to J.G.), and 4‐year Wellcome Trust PhD studentship in Developmental Mechanisms (to G.E.T.B.).

### Author's present address

N. Padmanabhan: Department of Cancer and Stem Cell Biology, Duke‐NUS Medical School, Singapore.
